# Meet the researchers: an alternative method of engaging patients with research in mesothelioma

**DOI:** 10.1186/s40900-018-0119-x

**Published:** 2018-10-15

**Authors:** Kate Hill, Mags Portman, Zsuzsanna Tabi

**Affiliations:** The June Hancock Mesothelioma Research Fund, c/o Irwin Mitchell, Riverside East, 2 Millsands, Sheffield, S3 8DT2 UK

**Keywords:** Patient and public engagement, Methods of engagement, Evaluation, Mesothelioma

## Abstract

**Plain English summary:**

There are new ways to engage people with science and research but many patient support groups and charitable organisations still hold traditional meetings to provide updates on their activities and to report new developments in their field of interest. These meetings often feature presentations given by medical doctors or, in the case of research-focussed organisations, by research scientists.

Receiving feedback from people who are confused and sometimes upset by some types of information, and the way it is presented at meetings, made us think about better ways for researchers to discuss their ideas for new research, or share the findings from completed projects, with patients and members of the public.

This article describes a method of public engagement called “Meet the Researchers” that enables people to hear about current trends in research face to face with the researchers planning or conducting it. “Meet the Researchers” is designed to promote discussion and allow questions to be asked in a relaxed and informal way, in small groups, which is less daunting than asking questions in front of a conference audience. The aim is to break down the barriers between researchers and patients, and enable conversations that will lead to meaningful engagement and a better understanding of research. Additionally we aim to improve understanding of how results are passed on to doctors and nurses and translated into improvements in patient care.

The method was tested with patients and was rated very highly by them in the feedback they gave.

**Abstract:**

**Background**

Innovative approaches to engaging people with science exist but are often framed around interactive events or social media technologies. Notwithstanding the availability of novel approaches, many patient support groups and charitable organisations continue to hold traditional meetings and seminars to provide information and updates on their activities, and report on developments in their field of interest. In the case of research-focussed organisations, these meetings often take the form of presentations delivered by clinical experts or research scientists.

Observation of mesothelioma patients, their relatives, friends and carers attending scientific or clinical-themed meetings has shown that they can be confused, and sometimes distressed, by presentations. This can be due to didactic presentations that are not properly targeted to this audience and a lack of a general overview or summary at the end of meetings that would provide some simple take home messages. This experience motivated the development of a less formal method of sharing complex information and ideas in a simplified manner. “Meet the Researchers” aims to make researchers accessible to patients in order to raise awareness and understanding of research and to explain how research translates into, and informs practice. This approach encourages the use of plain English, removes the tendency to rely on PowerPoint slides to convey the message and moreover, provides an opportunity for researchers to hear patients’ views.

**Methods**

Small groups of participants met face to face with the researchers planning or conducting research into their condition, and discussed the topics in a relaxed and informal way. The researchers spent a minimum of 20-min with each group before moving on to the next. Info-graphics on a portable device or printed hand-outs in plain English were allowed but no formal presentations were made.

**Results**

Our method has been evaluated using feedback data from three annual events held from 2016 to 2018: 100% of participants indicated that they liked the format “very much”(76.0%) or “quite a lot”(24.0%); 80.4% found the topics “very interesting” and 75.9% found it “very easy” to ask questions. Free text comments revealed themes of ‘hope’ and ‘altruism’. Researchers also reported benefits from participation such as learning about patient’ priorities and networking.

**Conclusion**

“Meet the Researchers” provides a unique opportunity for mesothelioma researchers and patients, relatives and carers to interact on a more equal footing. It stimulates discussion, promotes understanding and provides a more informal setting for non-professional participants to ask questions. It is a format that could easily be adapted for use in other conditions.

## Background

Mesothelioma is an asbestos-related cancer, usually but not exclusively, caused by occupational exposure. It is classified as a rare disease, and it is recognised that patients with rare diseases can be important partners in research [1]. Mesothelioma has a long latency period and is usually diagnosed when the patient becomes symptomatic; at this stage treatment options are limited, and prognosis can be short, hence the psychological burden of the disease is high. These factors limit the willingness and the capability of patients to become actively involved in research. Building a research portfolio that reflects the needs and aspirations of mesothelioma patients can therefore be challenging.

### Framing the context of engagement

Defining “patient engagement” is not straightforward; the literature on the topic is extensive but muddled because the terms “involvement” and “engagement” are used interchangeably. A qualitative study and systematic review concluded that while common concepts existed, the lack of clear terminology and definitions create ambiguity and confusion among stakeholders when referring to patient engagement [2]. Angela Coulter’s widely used definition of engagement focuses on the relationship between patients and health care providers working together to “*promote and support active patient and public involvement in health and healthcare and to strengthen their influence on healthcare decisions, at both the individual and collective levels.*” [3] For the purpose of this paper, and because our focus is on patient engagement with research, we have chosen to adopt the definitions proposed by INVOLVE [4]:

“*Patient engagement is where information and knowledge about research is provided and disseminated.”*

This is distinct from their definition of involvement: “*Where members of the public are actively involved in research projects and in research organisations.”*

Innovative approaches to engaging people with science are often framed around technology or social media; for example the #whywedoresearch campaign [5]. Many museums, art galleries and other special-interest centres have interactive displays and activity centres where people can see how things work and try experiments for themselves or practice their own creative skills. Additionally, the Velindre Cancer Centre in Cardiff [6] and other NHS organisations organise laboratory visits in conjunction with charities like Cancer Research UK [7] and Cancer Research Wales [8]. Science and research roadshows are also popular and just three examples of successful approaches are as follow: Southampton University’s Roadshow [9], the CHaOS Roadshow [10] and the National Institute of Health Research “I am Research” event [11]. Closer to the method we describe in this paper (but not identical) is the National Cancer Research Institute’s “Meet the Expert” session [12], which is held at their annual conference. These are informal sessions during which attendees have an opportunity to meet an eminent researcher in their field (typically a plenary speaker presenting at the Conference) and ask questions.

Notwithstanding these novel approaches, many patient support groups and charitable organisations continue to hold traditional meetings and seminars to provide information and updates on their activities and report on developments in their field of interest. In the case of research-focussed organisations, these meetings often take the form of presentations delivered by clinical experts or research scientists. Improving methods of engagement with researchers is a first step towards developing partnership in priority setting and coproduction of research [13] but evidence for the best way of achieving it is scant [14].

In this methodological paper we describe a successful approach to patient engagement with research in mesothelioma. The aims of this method of patient engagement were threefold:

1. To provide information about research in lay terms (Researcher to Patient)
2. To break down barriers, and provide an informal question time for patients and carers
3. To provide feedback (Patients to Researchers) about their perception of research and areas of research needs.

An evaluation of the method is also reported based on the feedback received from patients, relatives and professionals attending the last three such annual charity events.

## Methods

The “Meet the Researchers” concept is a fusion of speed-dating (where a group of singles meet for a fleeting date with each other, typically of 3 to 4 min duration) and the Dragon’s Den (a popular TV series in which budding entrepreneurs present their ideas to a panel of investors). The idea was driven by observations of patients’ at conventional meetings, and the need to move away from a format in which professionals ‘address’ the audience, and create an environment more conducive to disseminating information through discourse and questions. “Meet the Researchers” was designed to offer participants the opportunity to hear about current trends in research in small groups, face to face with the researchers planning or conducting it, and enable a discussion to flow in a relaxed and informal way. The aim was to make researchers accessible to patients in order to raise awareness and understanding of research and to explain how research informs and translates into practice.

The “Meet the Researchers” format was first enacted in a mesothelioma patient group at the June Hancock Mesothelioma Research Fund (JHMRF) Action Mesothelioma Day Event in Leeds in 2011 [15]. Action Mesothelioma Day is a national event that takes place annually on the first Friday of July. Mesothelioma charities, as well as local asbestos and mesothelioma support groups, organise public meetings in different locations. The JHMRF is a charity that relies solely on donations from the public. It is run by volunteers and has no paid employees. The JHMRF Action Mesothelioma Day “Meet the Researchers” event is the charity’s annual public facing event; it is free to attend. Lunch and refreshments are provided but participants are not reimbursed for attending. Participants include mesothelioma patients, their friends and relatives; bereaved relatives; representatives from other local cancer support groups, healthcare and legal professionals. A breakdown of participants who completed feedback forms in the three years from 2016 to 2018 is shown in Table 1.

**Table 1:** Participants’ profile 2016–2018

Participant Groups*	2016	2017	2018	Total
Patients	14	8	17	39
Relatives/carers/ bereaved relatives	18	14	16	48
Healthcare and other professionals	8	2	5	15
Total providing feedback	40	24	38	102
Percentage of total attendees	47.6%	35.8%	51.4%	45.3%

**Fig. 1:**
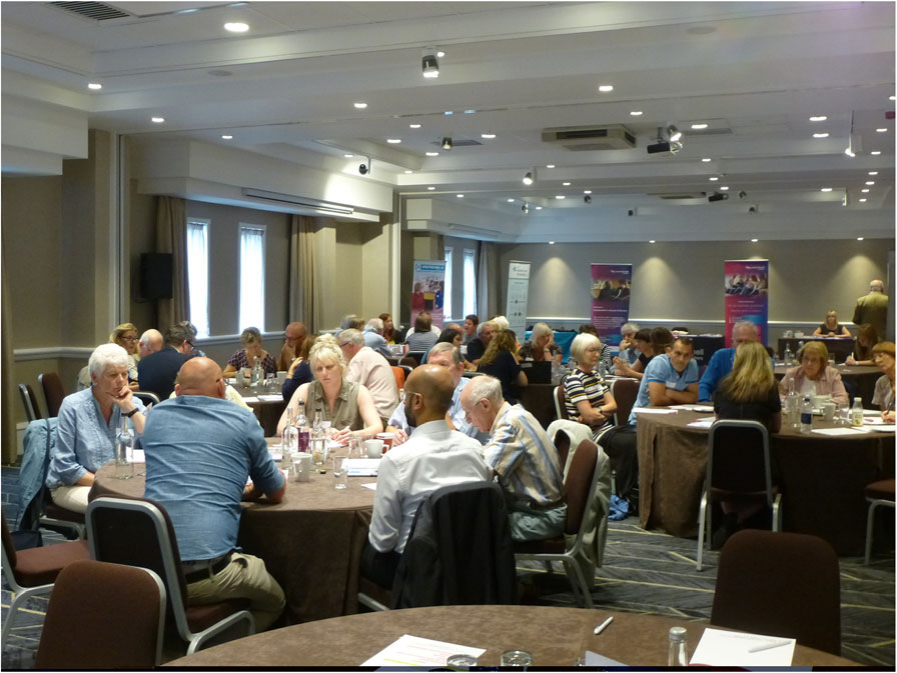
Room Layout

A large room with circulation space was used and laid out in cabaret format: circular tables of 8 to 10 (see Fig. 1). The tables were numbered (1 to n) and two spare seats were provided at each table. Patient participants were asked not to change tables during the day. A short introduction was given to brief participants about the research groups attending, and how the meeting would be conducted.

**Fig. 2:**
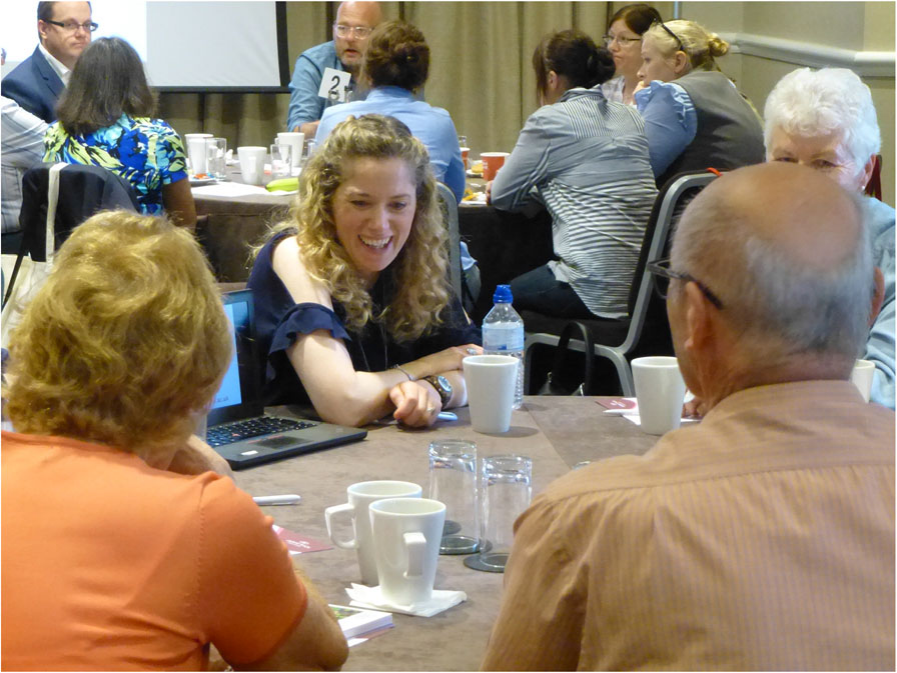
Table session in progress

The researchers were allowed a minimum of 20-min at each table (timing can be flexible depending on the meeting schedule but it is important to allow time for breaks, ideally after each round of no more than three table sessions, and for summing up at the end). Each research group (1 or 2 representatives) was asked to prepare a brief introduction for a non-professional audience that could be delivered in a maximum of 5-min. This was the ‘Dragon’s Den’ pitch, which outlined research interests or a specific research project; the remainder of the table session (15-min) was open for the ‘speed-dating’ questions from the table (see Fig. 2). Aids to understanding in the form of infographics or printed handouts in plain English were permitted, and a tablet or lap-top could be used to show one or two pictures, graphs or diagrams either as part of the opening pitch or to illustrate points raised in the ensuing discussion.

The research groups moved sequentially from table to table. Group 1 started at Table 1 and progressed round the remaining tables to finish at Table 8; Group 2 started at Table 2 and moved round to finish at Table 1 and so on. Flexibility is required in forming research groups and tables depending upon the number of attendees, and availability of researchers. The options for group configuration are either by specific project groups or by research discipline. Table 2 shows the research groups participating in Action Mesothelioma Day 2017 as an example of the areas covered.

**Table 2:** Research Groups 2017

Group 1	Organic chemistry	JHMRF PhD Fellowship: A structure-activity study of JBIR-23 to determine the components required for activity against mesothelioma cell lines.
Group 2	Patient Research Ambassador	Patient and Public Involvement in research
Group 3	Clinical research	JHMRF funded project: SYSTEMS 2 A trial of radiotherapy for pain control in mesothelioma.
Group 4	Surgical research	MARS2: A feasibility study comparing (Extended) Pleurectomy Decortication versus no Pleurectomy Decortication in patients with mesothelioma. Funded by Cancer Research UK and Papworth Hospital NHS Foundation Trust.
Group 5	Thoracic Oncology	Immunotherapy of MPM by blockade of suppressor intratumoural Treg: target identification
Group 6	Cell Biology	JHMRF PhD Fellowship: Understanding the pathogenesis of mesothelioma.
Group 7	Applied Research	RADIOMESO: Receiving a diagnosis of mesothelioma: improving the patient experience. Funded by Mesothelioma UK

Table moderation is the role of the facilitator assigned to each table; this is a key role (suitable for a Trustee or charity associate with the right skill set). The facilitator assists with time keeping and eliciting or moderating the questions. They also keep notes for the summing up at the end of the event, although another person can be nominated by the table members to deliver the feedback.

Three to five minutes before each table session ended, an amber warning card was shown to allow groups to wind down the discussion. At 20-min an audible warning and red card signalled time to move on to the next table. It is very important to keep to time; if a discussion was unfinished or questions unanswered the table facilitator noted them so that unresolved issues could be dealt with in the breaks or during the summing up session. The research groups are required to move on to the next table to avoid disrupting the flow and delaying the schedule. It is also important to encourage attendees to remain with the same table group throughout the meeting to ensure they have the opportunity to meet all the research groups.

## Results

### Evaluation

The JHMRF has used the “Meet the Researchers” format successfully for annual Action Mesothelioma Day events from 2011 to the present. Feedback has been evaluated using two types of data: quantitative descriptive statistics derived from a standard questionnaire, and qualitative comments derived from the free text sections of the questionnaire and from email messages, texts and thank you letters sent by participants after the event.

The “Meet the Researchers” format has proved to be extremely popular with people attending Action Mesothelioma Day events compared to previous events (held from 2008 to 2011), that used a traditional format featuring invited speakers and formal presentations. We acknowledge that feedback data is generally skewed to the positive but feedback for “Meet the Researcher” events has been much improved both in terms of the number of forms completed, and the number and content of comments. Combining data from 2011 to 2018, the proportion of feedback forms completed by attendees (*n* = 379) was 42.0%, of which about a third were patients and more than half were relatives and carers. Less than 10.0% of participants provided feedback in the years preceding 2011.

We aggregated our feedback data for the years 2016 and 2018. Rated on a four-point categorical scale: “*not at all”; “a little”; “quite a lot” and “very much”,* 76.0% of respondents liked the format *“very much”* and 24.0% liked it *“quite a lot”*. On a similar scale, 80.4% of respondents rated the topics covered as *“very interesting”*. The question relating to presentation of information proved more discriminating and indicates that, although the majority of respondents found the information presented *“very clear and easy to understand”* (65.3%) or *“quite easy”* (25.3%), some still struggled: 8.4% of respondents rated the information they received as *“moderately easy to understand”* and one respondent found it *“not at all easy to understand”*. This finding supports the evidence on which the “Meet the Researchers” model is predicated: that giving information about research to patients and carers cannot be construed as a simple and straightforward undertaking. Nevertheless, 75.9% of respondents found it *“very easy”* to ask the questions they wanted to ask, and this is reassuring as it suggests that the “Meet the Researchers” format does indeed facilitate interactive discussion and enables participants to ask questions more easily.

Feedback from JHMRF Action Mesothelioma Day events held before 2011 focussed on practical aspects of the meeting like the venue and catering. Consequently suitable data from the events we held using a traditional format is not available to compare with our feedback from subsequent “Meet the Researchers” events.

### Qualitative data

Space was provided on the feedback forms for respondents to enter any comments about the day. While not everyone took the opportunity to express their views, a surprising number of comments were received on the forms and many verbal comments were made to, and noted by, table facilitators. Many thank you letters, text and email messages were also received after the meetings. The comments from all years were collated and three broad categories emerged:

1. Practical aspects of the meeting
2. The Future
3. Information

#### Practical aspects of the meeting

Problems experienced at the meetings, expression of thanks and appreciation, and suggestions for future events were the themes associated with the practical aspects of the meeting. Problems identified included noise from adjacent tables, difficulty hearing and fatigue. Noise, occurring as a result of many conversations taking place simultaneously, was a common complaint across all years despite an attempt to address the problem by changing the venue for a larger room. Increasing the distance between the tables, albeit at the expense of some exhibition space, proved to be effective. We did consider using breakout rooms but the short sessions are not conducive to moving between rooms; moreover the cost to the charity would be prohibitive as we would need a room for each “table” of participants in addition to a large room for the collective sessions.

Suggestions from participants for future events were mostly practical hints and included allowing 5-min at the end of each table session to confer among themselves about points requiring further clarification or to formulate additional questions for the summing up session.

#### The future

Within The Future category were themes of hope and altruism linked with sentiments such as ‘helping others’ and ‘leaving a legacy’. Examples of comments included:

*“Just being in front of a researcher gives me some hope – if not for me, for others in the future”* and *“It’s great to know that not everything is doom and gloom – that there is hope - that research is going on and that our input today may help researchers help mesothelioma patients even more.”*

#### Information

Information was a broad category within which many cross-cutting themes emerged. Many respondents described the day as *“informative”* or said that they *“felt better informed”* but were not explicit about how they had been informed; these comments were frequently linked to expressions of thanks and appreciation. A few respondents were more specific about the information they had received, for example

*“To be up close with the researchers was invaluable, to know what is going on behind the scenes is reassuring. Lovely, informal, informative day.”*

*“I found the talk on radiotherapy for the new planned treatment for pain in meso patients very informative, and the fact that it will be available at (hospital named) soon”.*

At our most recent event we tried to tease out why attendees felt better informed by specifically asking if attendees had found the meeting useful, and providing a free text space for them to comment on this aspect. All respondents (100% in 2018) indicated that they had found the meeting useful, and several comments were received. The following extracts capture the three main categories that emerged from the free text data:

*“It gave information on subjects we find difficult.”*

*“Gave a deeper understanding of research.”*

*“Keeps me up to date with research and developments.”*

### The professionals’ perspective

We asked our researchers for feedback on the method too; some completed standard feedback forms while others preferred to send comments by email after the meeting. We found that “Meet the Researchers” posed challenges for some researchers, especially for those who had no patient-contact before or who had always used formal PowerPoint presentations in the past. This new format of meeting gave them the opportunity to develop and practice their communication skills to a largely non-professional audience, and it was perceived as a positive experience. Nearly all cancer research grant applications now request a plain English summary and the Action Mesothelioma Day meeting is an ideal forum to present ideas or results to a general audience. Meeting the sufferers of the disease can also be a humbling experience for those who are not clinical researchers. A young laboratory scientist attending the event for the first time commented:

*“It was a very valuable experience for me. I've never had any form of patient contact before so there was a lot I took away from the day, and I had lots of feedback to give our team…”*

Another young clinical researcher commented:

“*Today has be a salutary reminder of why we do research and who for….”*

Even more experienced researchers felt the event was worthwhile, a research group leader commented after the first event in 2011:

“*It was a very interesting new format as neither of us has been at a “speed dating” before! ...It worked extremely well, as people who otherwise would not have asked any questions were more confident in a small group setting to actively participate.”*

Another commented that it was

“*A worthwhile and educational experience for all”.*

The informal nature of the meeting also facilitates interaction and informal talks between participating scientists, working on wide-ranging aspects of mesothelioma.

A full evaluation report of the feedback from Action Mesothelioma Day 2016 and 2017, including quantitative data tables and a full list of free text comments, is available on the JHMRF website [13].

## Discussion

### The challenge of patient engagement in mesothelioma

First-hand experience of the difficulties faced by researchers in communicating complex research ideas and results to patients with mesothelioma led us to reflect on approaches to dissemination to this patient group. Two examples, in particular, come to mind: first, watching patients bury their heads in their hands or become tearful when presenters show (not very optimistic) survival curves, or describe symptoms like difficult pain and breathlessness; and second, witnessing audiences in the afternoon sessions of full-day events stare, silently and glassy-eyed, at yet another presentation with numerous slides showing complicated tables and images. The most compelling observation, however, was an encounter with a patient who left one of these meetings abruptly, saying: “*I’m sorry, I just have to escape – I can’t take any more of this……it’s all doom and gloom*.” This was a timely and cogent incentive for us to think more creatively about how we give patients information, especially those with terminal conditions.

Patient engagement in research is now an essential requirement for research grant applications to core funders like the National Institute for Health Research (NIHR) [16] and the Medical Research Council (MRC) [17]. INVOLVE was established in 1996 [18], funded by the NIHR, to support active public involvement in NHS, public health and social care research; and UK Research and Innovation (UKRI) recently published a concordat for patient engagement in 2018 [19]. As a research-funder, the JHMRF is also keen to build a research portfolio that incorporates the views, and reflects the needs, of mesothelioma patients and those close to them. Nevertheless, involvement in research priority setting places a burden of responsibility on predominantly lay people, with varying levels of experience and preparedness for the role, at a difficult time; and it is unrealistic to expect ordinary members of the public to become consultants in research design and collaborators in the process of the research; or shapers of health care policy without helping them to acquire the knowledge and skills they need to become actively and meaningfully involved. “Meet the Researchers” is designed to build rapport between patients and researchers thereby creating opportunities for patients to shape the JHMRF research agenda in a way that is less demanding for them. By using the football analogy of red and yellow cards, and the whistle to signal time, we aimed to reduce the formality of the meeting and relax participants by adding a little fun to the event. The short, interactive sessions diminish meeting fatigue and allow researchers and patients, relatives and carers to interact on a more equal footing.

### Methods of engagement

“Meet the Researcher” (or Meet the Experts) events are held in other settings but typically these are themed around a single expert or a panel of experts, speaking with an audience [12, 20]. The NCRI’s Dragons’ Den Workshop [21] is the closest methodological comparator as it offers the opportunity for researchers to discuss ideas with, or pitch research projects, to small panels of patients, corresponding to our table sessions at our “Meet the Researchers” event. The major difference is that the NCRI Dragons’ Den is focussed on partnership, problem-solving and co-production in relation to research proposals. “Meet the Researchers” is not designed to appraise research but aims to raise patients’ awareness of research in mesothelioma and help them to understand the way research is funded, conducted, reported and eventually translated into practice. An unanticipated outcome from our qualitative evaluation of the method was the extent to which researchers reported learning from the event. This supports the argument postulated by Staley (2017) that we should rethink our definition of impact when evaluating patient engagement and involvement activities [22].

The “Meet the Researchers” method of engagement is not limited exclusively to research. For example, if the meeting focus is on care, “Meet the Experts” could be conducted in a similar way with medical specialists, specialist nurses, allied healthcare professionals from different disciplines or medico-legal experts forming the expert groups. In this example, however, it is important for the table facilitator to moderate the table conversations effectively, and prevent individual cases becoming predominant. This was a problem we encountered when we mixed research and clinical experts for our “Meet the Experts” event in 2015.

Although we have not used our method for more targeted patient involvement activities, we speculate that it could also be applied to identify patients’ priorities for research, or elicit patients’ views on study design when planning a new project. A 2014 review by Brett et al. reported that lack of preparation and training led some service users to feel unable to contribute to the research, while other service users and communities reported feeling overburdened with the work involved. Researchers reported difficulties in incorporating patient and public involvement (PPI) in meaningful ways due to lack of money and time [23]. “Meet the Researchers could potentially overcome some of these difficulties by linking researchers with a large number of patients, without the time or expense of separate meetings or by replacing reviews of lengthy, written research proposals with group discussions, thereby reducing the workload for patients.

### Limitations of the method and evaluation

There are some aspects of our method that require refinement; for example providing additional breaks during the sessions to reduce fatigue (both patients and experts) and managing the noise level, which we overcame in 2018 by increasing the distance between the tables albeit at the expense of exhibition space for our charity and our partners in the event. Feedback from all participants also suggested that better briefing of researchers and table facilitators is required to ensure that information is presented, and questions are answered, in a clear and accessible way. Moreover, feedback from our 2017 event indicated that a brief outline of researchers and their field of expertise would be valued by both professional and non-professional participants. This would enable table facilitators and attendees to prepare questions, and researchers to cross-reference each other’s work to link up discussions more effectively and not appear as isolated, unconnected examples coming from individual laboratories. This latter suggestion was implemented at our most recent (2018) event and was well received; evidenced by the fact that not a single programme was left behind at the end of the meeting.

We acknowledge that our evaluation is constrained by the nature of the data collected from feedback forms. We also recognise that assessing the impact and benefit of engagement activities for patients is difficult because methods of evaluation are under-developed and evidence is limited [24]. Reports of similar events are generally found only in organisations’ newsletters and on their websites; and our scoping review revealed only one published paper reporting an evaluation of event feedback [25]. This event (a national PPI day for thyroid eye disease) used a combination of approaches to engagement, including didactic lectures, and focus group discussions. Fifty-two percent of attendees at the event provided feedback and of these respondents, 88% rated it very good or excellent. This is comparable with the feedback we received at our “Meet the Researchers” event but it is not possible to comment on whether the combination of lectures and focus groups is better than our informal discussion-based approach due to the limitations of feedback data. Options for wider comparison of our results are limited because reviews of patient engagement activities tend to focus on the impact, not the method, of engagement on research or practice.

## Conclusion

“Meet the Researchers” is a method of public engagement that provides a unique opportunity for mesothelioma researchers and patients, relatives and carers to hear about current trends in research face to face with the researchers planning or conducting it. The informal approach breaks down the barriers between researchers and patients and enables interaction on a more equal footing, without the use of PowerPoint presentations [26]. This reduces the impression that researchers are “talking to” the participants and helps stimulate conversations that lead to meaningful engagement, and a better understanding of research.

Feedback from our Action Mesothelioma Days has shown that participants felt empowered to ask questions because they found it less daunting to speak in small groups than they would in front of a conference audience. They also reported feeling better informed after the event and, importantly, said they felt that their experiences and opinions were valued. This is an encouraging outcome because being better informed and feeling able to contribute are springboards to future involvement [27]. Moreover, the participating researchers said they had benefitted and this too has important implications for facilitating future research collaborations and co-production.

The practical details included in the paper will be useful to the organisers of patient engagement events, particularly those seeking a new approach. The evaluation of the feedback provides some insight into the application of the method and how it is received by participants. The “Meet the Researchers” method could easily be replicated or adapted for use in other conditions and settings. It is also flexible and can focus on one theme or cover a range of topics from basic science to clinical trials and health services research. In this way, patients can be supported to recognise the different types and stages of research, and understand how results are passed on to doctors and nurses and translated into improvements in patient care.

## Abbreviations

ELF: The European Lung Foundation
JHMRF: The June Hancock Mesothelioma Research Fund
MRC: Medical Research Council
NCRI: National Cancer Research Institute
NHS: National Health Service
NIHR: National Institute for Health Research
PPI: Patient and Public Involvement
UKRI: UK Research and Innovation
